# Reduction in Substance-Related Composite Harm Scores Through Street Soccer

**DOI:** 10.7759/cureus.39650

**Published:** 2023-05-29

**Authors:** Alan T Bates, Lurdes Tse-Agha, Arun Agha, John-Jose Nunez, Heidi N Boyda, Andrea A Jones, Alasdair M Barr, William G Honer, Fidel Vila-Rodriguez

**Affiliations:** 1 Psychiatry, The University of British Columbia, Vancouver, CAN; 2 Psychiatry, BC Cancer, Vancouver, CAN; 3 Endocrinology, Queen's University, Kingston, CAN; 4 Palliative Medicine, Queen's University, Kingston, CAN; 5 Pharmacology and Therapeutics, The University of British Columbia, Vancouver, CAN; 6 Neurology, The University of British Columbia, Vancouver, CAN

**Keywords:** psychiatry and mental health, soccer, street soccer, physical fitness, sport activity, homelessness, addictions

## Abstract

Introduction

Street soccer makes the sport accessible to people affected by homelessness or precarious housing. There is overwhelming evidence that exercise improves physical and mental health. In addition, sport facilitates positive peer pressure that leads to beneficial life changes.

Method

To examine participants’ accounts of the effects of street soccer in a sample of socially disadvantaged players from Western Canada, we collected 73 cross-sectional self-reports of life changes via a questionnaire. The questionnaire included questions on social, mental, and physical health, including substance use. This allowed the calculation of a modified composite harm score.

Results

Participants reported improved physical (46% of participants) and mental (43% of participants) health, reduced cigarette (50% of smokers), alcohol (45% of users), cannabis (42% of users), and other non-prescribed drug use, increased number of friends (88% of participants), improved housing (60% of participants), increased income (19% of participants), increased community medical supports (40% of participants), and decreased conflicts with police (47% of those with prior recent conflict). Perceived reductions in substance use were supported by significant changes in composite harm score.

Conclusion

Street soccer appears to promote improved physical, mental, and social health among people affected by homelessness or precarious housing, with reduction in substance use likely to be a key factor. This work builds upon past qualitative research showing the benefits of street soccer and supports future research which may help elucidate the mechanisms by which street soccer has beneficial effects.

## Introduction

A curious thing happened in Scotland in the late 1970s and early 1980s. Every four years saw an eight-week period where psychiatric emergency presentations fell by 7-17%, with the effect being particularly robust in males. This was not the result of a well-orchestrated public health campaign, but rather of the Scottish National Soccer Team qualifying for the World Cup [1]. The authors who reported the finding suggested soccer had provided a “focus of purpose and excitement.” While healthcare providers cannot take credit for the fortunes of international soccer teams, creative health programs have attempted to engage people in prevention and treatment through the appeal of the world’s most popular game. Pringle and Sayers, for example, established a psychoeducation program for guys concentrating on mental health awareness in a professional football stadium [2]. Group members were “players” on a “team” who signed a “contract” at the beginning of the course of group sessions that comprised the “season.” Participants felt the soccer theme was engaging, with one commenting that it reduced the stigma of getting help by making it more of a “blokey thing.” Given all the positive health effects associated with physical fitness, one has to wonder if these men would have benefitted even more if they had actually played on a team together.

Evidence of the positive effects of exercise on physical health is overwhelming. As reviewed by Diehl and Choi, the benefits of regular physical activity include decreased risk of obesity, cardiovascular disease, diabetes, cancer, and osteoarthritis [3]. People with mental illness are at even greater risk of developing these chronic physical health problems, e.g., cardiovascular disease and diabetes [4,5], and unfortunately are also more likely to engage in activities that increase risk (e.g., smoking) and less likely to exercise regularly [6]. Psychiatric medications can also increase the risk of physical illness. For example, atypical antipsychotics predispose people to metabolic syndrome [7]. Fortunately, there is evidence that exercise can reduce that risk as well [8,9].

In addition to improving physical health, there is increasing evidence that physical fitness also improves mental health. Large epidemiological studies have shown a clear association between greater exercise and lower burden of mental illness [10,11]. While there is some question about the direction of causation in epidemiological studies [12], evidence increasingly suggests a bidirectional relationship [13]. Mounting evidence from interventional studies suggests a wide variety of exercise programs for people with mental illness are effective, including low-impact exercise such as walking [13] and yoga [14], resistance training [15], volleyball [16], moderate aerobic activity [17], and high-intensity interval training [18]. A meta-analysis of studies directly comparing exercise to antidepressants for treating depression found no significant difference in outcomes [19], while a Cochrane Review, using a meta-analysis, found the effect of exercise on depression was comparable to cognitive behavioral therapy in direct comparisons [20]. Recent systematic reviews and meta-analyses continue to show a benefit in depression, including in youth when taking into account publication bias [19,21], and when examining complementary outcomes to depression scores, such as quality of life [19], cognitive recovery [22], and neurobiological markers [23]. Exercise is also an effective intervention in anxiety and stress-related disorders [24,25], shows promise in attention deficit hyperactivity disorder (ADHD) [26], and may even be helpful in schizophrenia [27]. Neuroimaging data have shown increased hippocampal volume in people with schizophrenia after three months of aerobic exercise with increase in volume correlating with improved short-term memory [28].

A number of other marginalized or at-risk groups sharing similarities with people affected by severe and persistent mental illness also show benefits from exercise. Exercise appears to reduce depression in people with chronic physical illness (e.g., cancer, cardiovascular disease, chronic obstructive pulmonary disease, fibromyalgia, pain, HIV) [29-31]. The Special Olympics provide ample evidence of the benefits of sport for people with intellectual disability. For example, Özer et al. found decreased problem behaviors and increased social competence among youth with intellectual disability following participation in sport involving both healthy and intellectually disabled young people [32]. Sport is also highlighted as a behavioral strategy for preventing cravings among people with substance dependence [33-35]. Wynaden et al. found that a formal exercise program in a forensic mental health facility aided their participants in improving fitness, managing psychiatric symptoms, and building confidence and self-esteem [36].

Interventions seeking to increase physical activity in adults experiencing homelessness demonstrate a positive effect on activity levels [37] though there are also challenges in providing access and encouraging engagement in this and other hard-to-reach groups [38]. Team sports may offer opportunities for members to support each other as peers, which may be a pathway to improved outcomes as found in many types of interventions [39,40], including activities aiming to increase physical activity [41].

Street soccer is a low-barrier form of the game for people affected by homelessness, precarious housing, and related challenges [42]. Guided by abundant anecdotal evidence that street soccer players often achieve significant positive changes in physical and mental health, we hypothesized that such changes are associated with reductions in substance-related composite harm scores.

## Materials and methods

Study setting

A large portion of street soccer players in Vancouver is recruited from single-room occupancy hotels in a neighborhood known as the Downtown Eastside. Other studies carried out by our group in the neighborhood report mental illness (particularly psychosis), addictions, neurological disorders, and HIV and hepatitis C infections are common [43]. The Vancouver Street Soccer League (VSSL) has provided a number of opportunities for players including weekly practices, regular tournaments, games against the mayor, police, doctors, and medical students, and, for some, participation in the Homeless World Cup. VSSL players have played on Street Soccer Canada teams in Homeless World Cups in Milan, Rio de Janeiro, Paris, Mexico City, and Poznan. The VSSL’s activities have attracted the support of the Vancouver Whitecaps, Vancouver’s professional Major League Soccer (MLS) team, and attention from local (e.g., The Vancouver Sun), national (e.g., CTV), and even international (e.g., CNN) media.

Participants

Human participation in the study was approved by the Behavioral Research Ethics Board of The University of British Columbia, with approval number H11-00578. Anyone who identified as a street soccer player (and not a volunteer or organizer) was eligible to participate. Seventy-three participants were recruited at large street soccer tournaments in Vancouver and Kelowna, British Columbia.

Procedure

After giving informed consent, participants were asked to complete a self-report questionnaire asking about basic demographics, housing, substance use, physical health, mental health, perception of doctors, use of community health services and hospitals, conflicts with police, employment, social interactions, and reasons for playing street soccer. Several pairs of questions elicited a current rating of a variable (e.g., general health) and a rating for the same variable for the year before the participant started playing street soccer. Others asked participants to rate change in a variable since joining street soccer on a Likert scale (e.g., worsened a lot, worsened a little, not changed, improved a little, and improved a lot). For substance use, participants were asked to rate cigarette use per day as none, less than half a pack, half to one pack, about one pack, one to two packs, or more than two packs, and use of other non-prescribed substances as never, once per month, once every two weeks, weekly, a few times per week, or daily for two-time intervals (the past month and the year before joining soccer). Participants were allowed to complete the questionnaire verbally with the aid of one of the investigators if they were unable to complete it on their own.

Data analysis

Data were analyzed using Statistica 12 (Palo Alto, CA: TIBCO Software Inc.) and JMP (Cary, NC: SAS Institute). Null hypotheses related to distribution within Likert scales were tested using the chi-square test. For comparisons between time points, Likert scales were converted to ordinal ratings, and time points were compared using the Wilcoxon signed-rank test.

To better understand data related to substance use, we calculated a modified composite harm score (CHS) for each participant at each time point (past month and year prior to joining street soccer). The Independent Scientific Committee on Drugs (ISCD) has assigned harm scores to each of 20 different substances, including those that we asked about in this study, where each substance has a value between 0 and 100 based on its harm to users [44,45]. The CHS incorporates both the harm of each substance used as well as the frequency of use in a month. This score is associated with greater mortality, substance-induced psychosis, spending on non-prescribed substances, odds of committing a crime, and multimorbid illness. To calculate our modified CHS, we summed substance harm score × days of use per month over tobacco, alcohol, cannabis, methamphetamine, crack cocaine, powder cocaine, and heroin. We assigned descriptions of frequency of use of never, about once a month, about every second week, weekly, a few times per week, and daily to zero days, one day, two days, four days, 12 days, and 28 days per month. CHS comparisons between subgroups of participants were made using the Wilcoxon rank sum test and associations between CHS and other variables were carried out through ordinal logistic regression adjusted for age and sex.

## Results

Demographic information

Participant demographic information including age, sex, ethnic background, education, housing, and length of involvement in street soccer is presented in Table 1. Studies carried out by our group and others indicate that the profile is similar to the neighborhood overall [46,47]. Sixty-four percent of participants stated that they were unemployed. Reasons for unemployment are listed in Table 2, with half of those unemployed noting illness or disability as the primary reason. We also inquired as to health factors that may contribute to them not working. Forty-one participants (56%) stated that health concerns contributed to their unemployment, including concerns related to physical health (11 participants, 15%), emotional or mental health (25 participants, 35%), or substance use (eight participants, 11%).

**Table 1:** Demographics of the street soccer participants in our study.

Demographic		Values
Mean age (n=71)		32.4 (range: 17-60)
Female, male (n=72)		20 (28%), 52 (72%)
Ethnic background (n=73)	Caucasian	30 (41%)
	Indigenous	24 (33%)
	Black Canadian	7 (10%)
	Latin American	6 (8%)
	Chinese	2 (3%)
	Southeast Asian	2 (3%)
	Filipino	1 (1%)
	Korean	1 (1%)
Education (n=72)	No high school	2 (3%)
	Some high school	25 (35%)
	High school graduate	24 (33%)
	Some college/university	7 (10%)
	College/university graduate	14 (19%)
Housing (n=73)	Self-contained apartment	25 (34%)
	Single-room occupancy hotel	13 (18%)
	Shelter	11 (15%)
	Rented room in a house	8 (11%)
	None/on the street	4 (5%)
	Recovery house/detox center	2 (3%)
	Other (e.g., trailer, parents’ home)	10 (14%)
Length of involvement in street soccer (n=73)	<1 month	18 (25%)
	1-3 months	10 (14%)
	4-6 months	9 (12%)
	6 months - 1 year	14 (19%)
	>1 year	22 (30%)

**Table 2:** Reasons for unemployment as provided by 48 of the participants.

Own illness or disability	24 (50%)
No work available	11 (23%)
Caring for own children	4 (8%)
School or educational leave	3 (6%)
Other personal or family responsibilities	1 (2%)
Vacation	1 (2%)
Retired	0 (0%)
Caring for elders	0 (0%)
Pregnancy	0 (0%)
Other	4 (8%)

General changes with street soccer

For the most part, participants agreed they enjoyed the physical exercise (63/72, 88%) and the company of teammates (65/71, 92%), and that street soccer provided a sense of routine (58/71, 82%) and self-pride (66/72, 92%). The majority of players also perceived social benefits. This included reporting improved housing, having more friends, and receiving more positive feedback from others as being associated with their participation in street soccer. A substantial proportion also noted increased income and decreased conflicts with police. These social benefits are summarized in Table 3.

**Table 3:** Social changes reported by the street soccer participants. *An additional 29 participants indicated no history of conflict with police.

Variables	Worsened	No change	Improved	p-Value
Housing (n=72)	1 (1%)	28 (39%)	43 (60%)	<0.001
Number of friends (n=73)	1 (1%)	8 (11%)	64 (88%)	<0.001
Positive feedback from others (n=71)	1 (1%)	7 (10%)	63 (89%)	<0.001
Income (n=68)	1 (1%)	54 (80%)	13 (19%)	<0.001
Frequency of conflict with police (n=38*)	1 (3%)	19 (50%)	18 (47%)	<0.001

Health changes with street soccer

**Figure 1:**
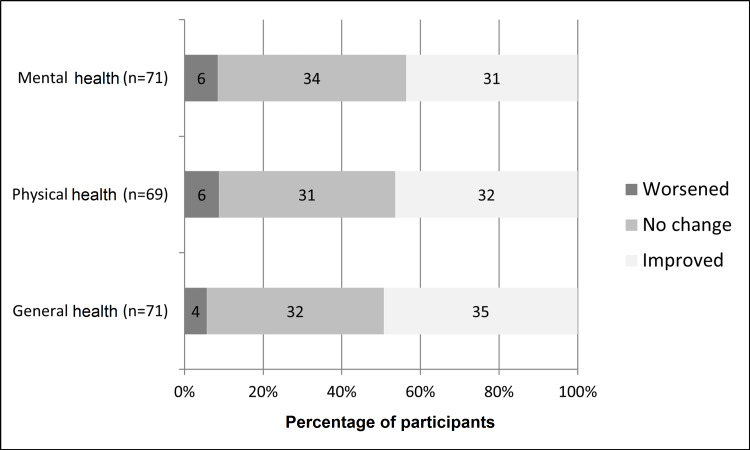
Perceived changes in health. Grayscale bars show whether players perceived worsening (dark), no change, or improvement (light) for each of the following: mental health, physical health, and general health. Numbers within the bars represent the numbers of participants and the cumulative percentage is indicated along the x-axis.

Participants were asked to rate their general, physical, and mental health currently and for the year before joining street soccer as either excellent, very good, good, fair, or poor. The majority of players rated their mental and physical health as good or better (Table 4). The vast majority of participants indicated each measure of health as either improved or unchanged over the two-time points (Figure 1). Twice as many participants rated their physical health as excellent over the past month (24/69, 35%) compared to the number of patients who rated it as excellent for the year before street soccer (12/69, 17%). After converting the Likert scales to ordinal values, mean improvements in descriptions of general health (0.86 {SD: 1.33}, z score=4.51, p<0.001), physical health (0.80 {SD: 1.38], z score=4.10, p<0.001), and mental health (0.68 {SD: 1.34}, z score=3.86, p<0.001) were statistically significant.

**Table 4:** Participant health ratings before and after street soccer participation, reporting general, physical, and mental health.

Variables	Poor	Fair	Good	Very good	Excellent	p-Value
General health before soccer (n=72)	17 (24%)	11 (5%)	16 (22%)	12 (17%)	16 (22%)	<0.001
General health past month (n=71)	1 (1%)	3 (4%)	22 (31%)	21 (30%)	24 (34%)	
Physical health before soccer (n=69)	13 (18%)	7 (10%)	21 (30%)	16 (23%)	12 (17%)	<0.001
Physical health past month (n=69)	1 (1%)	5 (7%)	18 (26%)	21 (30%)	24 (35%)	
Mental health before soccer (n=71)	15 (21%)	11 (15%)	10 (14%)	13 (18%)	22 (31%)	<0.001
Mental health past month (n=71)	3 (4%)	5 (7%)	17 (24%)	17 (24%)	29 (41%)	

Changes in substance use with street soccer

**Figure 2:**
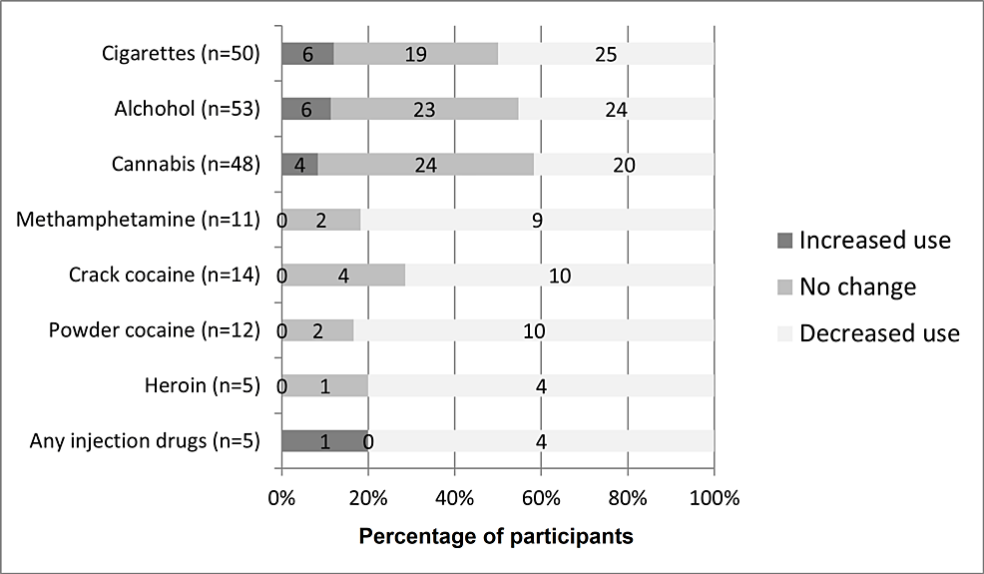
Perceived changes in substance use. Grayscale bars show whether players perceived increased use (dark), no change, or decreased use (light) for each substance. Only participants who indicated use of a substance either over the past month or during the year before engaging in street soccer are included. For each of the substances, many participants denied use during either of those time periods: cigarettes (22), alcohol (19), cannabis (25), methamphetamine (61), crack cocaine (57), powder cocaine (57), heroin (67), and any injection drugs (67). Numbers within the bars represent the numbers of participants and the cumulative percentage is indicated along the x-axis.

Figure 2 shows the pattern of reduction in most substances use. Very few participants endorsed the use of heroin or other injection drugs even before joining street soccer. Abstinence from alcohol showed a more than 50% increase (20-33 participants). About half of the participants using cigarettes (25/50, 50%), cannabis (20/48, 42%), or alcohol (24/53, 45%) reported decreased use. While fewer participants had a recent history of use of other substances, the vast majority of those with a recent history had reduced use of methamphetamine (9/11, 81%), crack cocaine (10/14, 71%), powder cocaine (10/12, 83%), heroin (4/5, 80%), or injection drugs in general (4/5, 80%).

A small number of participants reported increased use of cigarettes, cannabis, alcohol, and injection drugs (only one participant), but none reported increased cocaine or methamphetamine use. A closer examination of the data showed that increase in one substance tends to be associated with decrease in others. Of the six participants who increased the use of cigarettes, two decreased alcohol use, four decreased cannabis use, and one decreased methamphetamine use. Only one participant increased smoking without improvements in other forms of substance use. Of the six participants who increased the use of alcohol, three decreased cigarette use, one decreased cannabis use, four decreased methamphetamine use, and three decreased crack and/or powder cocaine use. Two participants increased alcohol use without a decrease in other substances. Of the four participants who increased the use of cannabis, two decreased cigarette use, two decreased alcohol use, one decreased methamphetamine use, and one decreased cocaine use. One participant increased cannabis use without decrease in other substances. The participant who reported increased use of IV drugs did not report reductions in the use of any other substances.

Composite harm scores

**Figure 3:**
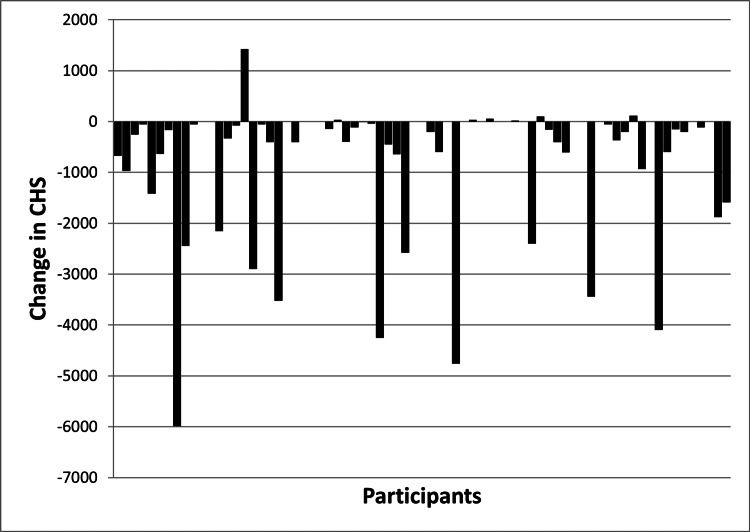
Histogram illustrating changes in modified composite harm score (CHS) for each participant. Each bar represents a single participant and the y-axis shows changes in CHS between the year prior to joining street soccer and the past month.

CHSs for the past month were significantly lower than CHSs for the year prior to joining street soccer (p<0.001). Figure 3 shows the change in CHS for each of the 68 participants. Higher CHS before participation in street soccer was associated with lower ratings of general health (p=0.0016), physical health (p=0.012), and mental health (p=0.005) before participation in street soccer, but CHS over the past month was not significantly associated with ratings of general health, physical health, or mental health over the past month. Decrease in CHS between the year before street soccer and the past month were associated with improved perception of general health (p=0.027), physical health (p=0.036), and mental health (p=0.0056) over the same time period. There was also a trend for reduction in CHS to be associated with length of involvement in street soccer of six months or greater (p=0.066). Participants whose housing improved over the course of participation in street soccer showed greater reduction in CHS compared to those who had no change in the quality of housing (p=0.009).

Changes in perception of healthcare

As summarized in Table 5, a statistically significant portion of participants reported improvement in their opinion of physicians (20 {29%} improved vs. 2 {3%} worsened). In addition, a significant portion reported their community-based medical support improved (25 {40%} improved vs. 1 {2%} worsened). Any change in the frequency of visits to hospitals was difficult to interpret as most participants indicated they had only been to a hospital once or not all over the year before joining street soccer.

**Table 5:** Changes in perception of healthcare. Changes in how street soccer participants perceive healthcare.

Variables	Worsened	No change	Improved	p-Value
Opinion of doctors (n=70)	2 (3%)	48 (69%)	20 (29%)	<0.001
Community-based medical support (n=63)	1 (2%)	37 (59%)	25 (40%)	<0.001

## Discussion

The findings suggest participation in street soccer is associated with improved physical and mental health that is accompanied by decrease in substance use and substance-related composite harm score. Improved housing, gaining friends, receiving more positive feedback, increased income, improved opinion of doctors, increased community-based medical supports, and decreased conflicts with police were also found. The results were in agreement with the changes we repeatedly observe as street soccer volunteers. They are also consistent with more qualitative results observed by others in people who have participated in the Homeless World Cup [48,49]. It appears that participation in sports has quite wide-ranging benefits for people affected by homelessness and inadequate housing. Perhaps the most remarkable aspect of this is that street soccer does not directly target the majority of the variables examined in the study. Rather, there seem to be several positive collateral effects of participation in sports.

How street soccer influences these positive changes is unclear. Improved physical fitness is one obvious candidate for mediating these changes. As outlined in the introduction, there is a wealth of evidence that physical fitness significantly impacts both physical and mental health. Randers et al. even demonstrated that 12 weeks of street soccer participation by homeless men increased maximal oxygen intake, and decreased body fat percentage, low-density lipoprotein cholesterol, and diastolic blood pressure [50]. However, there are other possible etiologies for street soccer’s success. Sport also provides increased social contacts and in the case of street soccer, players are connected not only with other players but also with volunteer organizers. In the case of the VSSL, several volunteers are well-connected with housing resources, health resources, or other community supports. Players often learn about community resources through informal conversations with volunteers and other players. Sherry suggests that street soccer increases the social capital of players by connecting them to individuals outside their immediate community and creating relationships that provide access to previously inaccessible resources [49]. Social capital is limited in this population but can support recovery from substance disorders and lead to other positive outcomes [51]. A similar phenomenon has been reported by Özer et al. who described how children participating in a Special Olympics soccer program benefited not only from playing with peers but also from playing with children without disabilities [32]. There's other evidence that modeling of appropriate social behavior in sports can translate into improved social behavior in other parts of life. For example, Rutten et al. found that both on-field and off-field antisocial behavior in adolescent soccer players could be predicted by variables including lack of support from their coach and lack of positive team attitude towards fair play [52]. Therefore, the efforts of street soccer coaches and other volunteers to be supportive, model positive behavior and attitude, and promote fair play could be influencing players' behavior off the field as well. Many of these factors may contribute to the decrease in substance use reported by the players, which could then be expected to feedback and further enhance some of these other mediators [44]. It may also be that reduction in substance use in order to perform better in sports is the core initial change that leads to improvements in other variables for the majority of participants.

The present study has a number of limitations. There was likely selection bias in terms of which players volunteered to fill out the questionnaires and which did not. The fact that the assessments of change were by self-report may also make the reliability of some of the findings more questionable. As an example, some street soccer players with significant mental illness do not have insight into the degree to which it affects them and this would have influenced their ratings of their own health. However, there is good evidence that self-reported measures of substance use correlate well with urine drug screen results in this population [45]. Though we attempted to gain information about different time points (the year before joining street soccer and the past month), it is still a cross-sectional evaluation possibly affected by recall bias, and more reliable comparisons might be made in a longitudinal study and by using a control. There was also some variability between participants with respect to their current housing (e.g., living in a shelter vs. single-room occupancy vs. living on the street) and the duration they had participated in the program. Particularly with participants who had only participated for a few months, it could be that participation in street soccer was part of a larger collection of positive changes being made rather than a factor with significant causal effect on other positive changes (e.g., reduced substance use). Further work with a larger sample may be able to further investigate how these differences impacted the studied outcomes. Despite these limitations, it seems clear that players believe their participation in street soccer improves their lives in a number of significant ways.

Homelessness and inadequate housing is an extremely complex problem and the positive findings of this study must be put in perspective. While street soccer does appear to have several beneficial effects, it is by no means a comprehensive solution. For a start, street soccer simply doesn't appeal to all people who are affected by homelessness or inadequate housing, and chronic physical illness or disability is a likely barrier for many. As noted by Magee and Jeanes, competitive sport is exclusionary by nature and street soccer does become more competitive at events such as the Homeless World Cup [46]. Even among those who participate, street soccer may not be a positive experience for everyone. At least one examination of player experiences at the Homeless World Cup highlights that some players feel some of their experiences at the tournament, such as poor performance or heavy losses, reaffirm feelings of failure [46].

## Conclusions

This cross-sectional study of 73 socially disadvantaged street soccer participants examined self-reported life changes by use of a questionnaire. Participants reported improved physical, social, and mental health, and a reduction in substance use, supporting the benefit of street soccer for those affected by homelessness or precarious housing. This work builds upon prior qualitative research showing the benefits of street soccer for health development, and upon research showing the health benefits of sports programming.

Future research may help elucidate the mechanisms by which street soccer has such broad beneficial effects on health and social outcomes. It’s likely these mechanisms could be applied to other sports and possibly even other non-athletic group activities that might have wider appeal to potential participants. We hope this work may advance the development of street soccer and other programs targeting the social, physical, and mental health of those excluded by traditional interventions.
